# Antibacterial Effects of Thermosonication Technology on *Salmonella typhimurium* Strains Identified from Swine Food Chain: An In Vitro Study

**DOI:** 10.3390/foods13203259

**Published:** 2024-10-13

**Authors:** Luca Pennisi, Gianluigi Ferri, Carlotta Lauteri, Daniele Di Clerico, Alberto Vergara

**Affiliations:** 1 Department of Veterinary Medicine, Post-Graduate Specialization School in Food Inspection “G. Tiecco”, University of Teramo, Strada Provinciale 18, 64100 Teramo, Italy; lmpennisi@unite.it (L.P.); clauteri@unite.it (C.L.); avergara@unite.it (A.V.); 2 Next Cooking Generation Srl, 20100 Milano, Italy; daniele.diclerico@ncg-tech.it

**Keywords:** thermosonication, cavitation phenomenon, *Salmonella* spp., bactericidal effect, food safety

## Abstract

Among innovative food technologies, ultrasounds have demonstrated physical damages (provided by frequency and intensity factors) on bacterial structures while determining the microbiological stabilization of many foodstuffs. This study tested the efficacy of the thermosonication process on 16 *Salmonella typhimurium* strains belonging to the academic biobank (isolated from swine slaughterhouses). All strains were exposed to focused ultrasounds, generated by the Waveco^®^ system (Milan, Italy), with the following settings: 40 KHz coupled with 80 W at different 5 min intervals starting from 5 to 15 ones, and focusing on two different temperatures: 40 °C and 50 °C. After each treatment, all strains were directly plated onto count agars immediately (t0) and after 24 h (t24) of storage at refrigerated temperature. The results showed bacterial reductions by prolonging the sonication treatments until 15 min (i.e., 50 °C for 15 min reduced of 2.16 log CFU/gr the initial loads). In the present in vitro study, the most considerable decrease was observed after 24 h. It meant that *Salmonella* strains were lethally damaged at the wall level, confirming the ultrasound bactericidal effect on loads. The present in vitro scientific investigation demonstrates the practical bactericidal effects of thermosonication, highlighting promising applications at the industry level for food microbial stabilization and shelf-life prolongation.

## 1. Introduction

Innovative and sustainable food technologies have gained more attention by the scientific community for their wide applicability in different productive sectors, with special regard to the so-called “*non thermal technologyies*” at the food industry level [1]. Among them, ultrasounds have demonstrated reduced impacts on food quality such as freshness, and also presenting bactericidal lethal and sublethal effects on the main pathogens (included in the EU Reg. No. 2073/2005 [2]), which could be normally harbored both in animal and vegetable origin foodstuffs [3]. In addition to the strict pathogens, focused ultrasounds revealed bactericidal activity also against commensal strains; their reduction, after exposures, determines the so-called shelf-life prolongation of foodstuffs. Indeed, this microbiological stabilization provides safe and quality products for final consumers [1].

Ultrasound is one of the non-thermal alternative food technologies useful for the overcoming of traditional ones [4]. Indeed, its efficacy is rooted in the physical activity covered by the acoustic cavitation phenomena which are responsible for lethal injuries on bacterial external structures [5]. In more detail, cavitation is a physical reaction that occurs when bubbles form, grow, and collapse in liquid media. This reaction has been shown to damage bacteria by altering cell morphology with a reduced thermal increase (ranging between 30 and 60 °C) differently to the traditional food technologies (e.g., pasteurization, boiling, etc.), as reported in several scientific investigations [6,7]. Nonthermal treatments generally cause sublethal injury states responsible for reduced cellular viability, which can lead to the apoptosis step; more specifically, sublethal injury (SI), also defined as the consequential exposure to a physical or chemical process, can kill microorganisms, as suggested by Hurst [8] and Wesche et al. [9]. The biochemical trigger for cellular death is represented by wall and/or membrane damages which are responsible for the genetic expression and synthesis of ligands (enzymatic molecules), leading to the auto-denaturation of the structural macromolecules (e.g., DNA, proteins, etc.) [7].

From an experimental perspective, the sublethal action of the cavitation process has been estimated as viable but non-culturable (VBNC), which refers to a state of cells that can be defined as an inactive form of life that is induced by stressful conditions, representing a direct diagnostic laboratory method to identify the inactivated strains [10].

Focusing the present reasoning on bacterial genera and species, different susceptibilities and responses among pathogenic microorganisms to the nonthermal treatments when exposed to similar physical setting conditions were observed (with special regard to the following bacteria, e.g., *Listeria monocitogenes* and *Staphylococcus aureus*) [11].

These natural and different behaviors, observed in the same genus, were scientifically identified and explained by Schimel et al. [12] and by Pavlov and Ehrenberg [13], who interpreted this phenomenon as an adaptation to the mechanical environmental stressors which act as codifying *stimuli* for specific genetic regions influencing their expressions [13]. It demonstrates that physical triggers can be considered highly influencing, as well as biochemical ones for bacterial genome codification [14,15]. This mechanism was also experimentally observed in *Escherichia coli* and *Staphylococcus aureus* strains exposed to sublethal ultrasound settings [16].

For this purpose, the present scientific in vitro study aims to test the lethal or sublethal effects (cellular injury) of thermosonication technology on 16 *Salmonella enterica* serovar *typhimurium* strains. The results regarding the specific effects on bacterial strict pathogens like *Salmonella* spp., included in the so-called *food safety criteria* and *food hygiene process* in the EU Reg. No. 2073/2005 [2], are poorly described in the scientific literature. Therefore, the obtained original data aim to contribute to a better understanding concerning the microbiological effects of mild food technologies on one of the main bacterial foodborne pathogens. Based on these considerations, it is mandatory to affirm that further scientific investigation results are necessary in order to contribute to a better understanding of the bacterial responses (which differ between Gram-positive and Gram-negative ones) to these food technologies. For this purpose, the present research intends to provide original data to enforce the consideration of ultrasounds as a widely applicable, innovative, and useful tool at the food industry level. Indeed, due to its efficacy on bacteria genera, both included in the EU Reg. No. 2073/2005 and commensal ones, it could contribute to the shelf-life prolongation of many food matrices.

## 2. Materials and Methods

### 2.1. Bacterial Strains

A total of 16 *Salmonella typhimurium* strains, isolated and bio molecularly identified from the swine slaughterhouse food chain (including fresh tissues and processed products, located in the Emilia-Romagna region, Italy), were involved in the present scientific study (as schematically illustrated in Table 1). They were stored at −80 °C by using cryovials (ROTI^®^Store cryo vials, Frankfurt, Germany), and are part of the academic biobank belonging to the Department of Veterinary Medicine (University of Teramo, Teramo, Italy).

As the first laboratory step, each cryo-conserved *Salmonella* strain was recovered and resuspended in 10 mL of Tryptic Soy Broth (Oxoid Thermo Fisher^TM^, Waltham, MA, USA) and incubated at 37 °C for 18–24 h. Solutions were considered useful for the other analytical steps when their respective turbidity ranged between 1.0 and 2.0 McFarland, measured using the nephelometer DensiCheck Densitometer (bioMérieux, Paris, France). Bacterial cells were then harvested by centrifugation at 3000× *g* × 15 min at refrigerated temperature, and successively washed three times with phosphate buffer saline (Liofilchem^®^, Roseto degli Abruzzi, Italy) 50 mM, pH 7.4.

Each inoculum quantification, for each tested *Salmonella* spp., was determined by the UV-Vis-NIR Spectrophotometer (Shimadzu, Kyoto, Japan) using the following standardization setting OD_620nm_ 0.1–0.2 (10 × 10^7^ cells/mL). This initial concentration was successively ten-fold diluted until 10 × 10^5^ cells/mL.

### 2.2. Ultrasound Treatments

Focused ultrasounds, used for the present scientific investigation, were produced using the Waveco^®^ system (Next Cooking Generation, Milan, Italy), which is an ultrasonic bath with a capability of 30 L (using distilled water for ultrasound transmission). The selected setting was 40 kHz with 100% amplitude, with a focused ultrasonic wave according to the patent (International Application No.: Patent EP17733039.6 [17]) and at 800 W. The temperature of the medium (distilled water) was monitored and controlled using a special construction system. The ultrasound transmission, through the liquid media, is responsible for the cavitation phenomenon, which lethally and sublethally injures the bacteria harbored in liquid and solid food matrices.

The above-mentioned setting (800 W/40 kHz/100% amplitude) was used to sonicate all specimens (10 mL: 9 mL PBS and 1 mL of 10 × 10^5^ *Salmonella* cells/mL or 5.0 log CFU/mL) at two different temperatures, 40 °C (T1) and 50 °C (T2), at different timing steps: 5, 10, and 15 min, respectively. All experiments were performed in triplicate including negative (sterile water) and positive (ATCC 14028 *Salmonella enterica* subsp. enterica (ex Kauffmann and Edwards) Le Minor and Popoff serovar *typhimurium*) controls.

### 2.3. Microbiological Analysis

All *Salmonella* strains used in the present study were isolated by using selective culture media such as the Rappaport-Vassiliadis Broth (ThermoFisher Scientific^TM^, Milan, Italy) and successive plating onto Xylose Lysine Deoxycholate (XLD) agar (Liofichem^®^, Roseto degli Abruzzi, Italy), in agreement with ISO 6579-1:2020 [18]. The identification process was performed using the VITEK^®^ 2 System (bioMérieux, Paris, France) and successively stored using cryovials (ROTI^®^Store cryo vials, Frankfurt, Germany) at −80 °C, as previously mentioned.

Before treatment, all cryo-stored samples were introduced in vials which contained 5 mL of Mueller Hinton Broth (ThermoFisher Scientific^TM^, Milan, Italy) and were incubated for 18–24 h at 37 °C. The viable strains were treated following the previously described settings and plated onto XLD agar, used as *Salmonella* spp. selective culture, and onto tryptic soy agar (TSA) one (Liofichem^®^, Roseto degli Abruzzi, Italy), used as the non-selective medium. In both cases, plates were incubated at 37 °C for 18–24 h. The double plating, as previously described, was performed at two different timing moments: immediately after thermosonication (t0) and after 24 h from treatment (t24), stored at refrigerated temperature. Positive and negative controls were always included.

*Salmonella* spp. counts were performed for all double-plated strains in the two described moments (t0 and t24), in agreement with the International standard ISO 6579-1:2020 [18]. All strains were double plated onto TSA and XLD, as suggested by Han et al. [19]; in more detail, the above-mentioned plating scheme could determine three different bacterial subpopulations, named as follows: healthy cells, sublethal injured (defined as exposure to a chemical or physical process that damages cells), and dead cells. It is also important to mention that sub-injured *Salmonella* strains easily grow onto a non-selective agar (TSA) than a selective one (XLD), permitting us to calculate the so-called *injury ratio* [20]. More specifically, the sublethal injured cells were estimated by the difference in the number of CFUs obtained after plating treated cells in the non-selective medium and the selective medium. The plates were incubated at 37 °C for 48 h. After incubation, the colonies were counted. Survival was expressed as the logarithmic viability reduction log10 (N/N0), with N0 and N representing the colony counts before and after thermosonication treatment, respectively. According to Bi and coworkers [20], the injury ratio was calculated with the following equation:

$$Injury\;ratio\left(\%\right)=100-\frac{CFU/ml\;non\;selective}{CFU/ml\;selective}\times 100$$

The range of responses to the focused ultrasound after t24 was evaluated with the following formula:

*CONTROL − TREATMENT*

The obtained values were interpreted as follows: values less than 1.0 log CFU/mL strain were considered resistant to treatment, between 1.0 and 2.0 log CFU/mL strains were considered intermedium, and strains were considered sensible if the obtained value was more than 2.0 log CFU/mL [19].

### 2.4. Statistical Analysis

The significancy effect of ultrasound parameters and the duration of the treatment on decreasing the number of *Salmonella typhimurium* were evaluated by performing one-way analysis of variance (ANOVA between subject) and the *t*-test applied with the Tukey-HSD test at the *p* < 0.05 significance level. Normality of variance was performed by using the Shapiro–Wilk test, and homogeneity of variances was tested by the Levene test (XLSTAT 2014 software, Redmond, WA, USA; Jamovi project 2022).

## 3. Results

**Figure 1:**
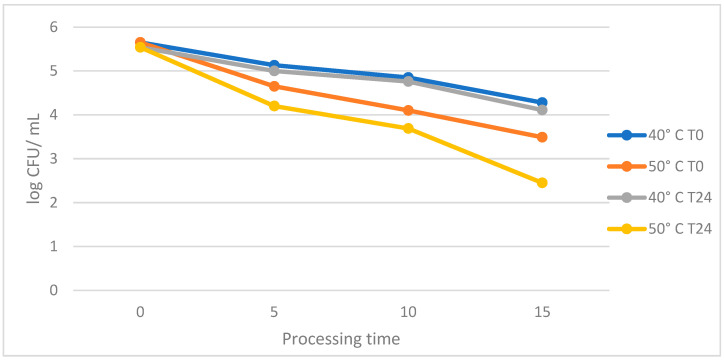
Effects on bacterial counts focusing on the ratio: temperature and processing times.

Starting from the standardized initial concentration (5.0 log CFU/mL), a consistent bactericidal effect (with an average reduction of 1.0 log CFU/mL) of focused thermosonication was observed at 40.0 °C and 50.0 °C after 5 min of exposure. Moreover, *Salmonella* strains, plated at t24 than t0, were injured, as represented in the following Figure 1. Indeed, when comparing the obtained log CFU/mL values between t0 and t24, significant statical differences were observed (*p*-values < 0.001) using the *t*-test.

The prolonged exposure at both temperatures (T1 and T2) showed an average reduction of about 1.5–2.0 log CFU/mL after 15 min with special regard to T2 in t24 and to a lesser degree than t0 (see Figure 1). The best effective treatment was 15 min at T2 in an in vitro assay, where the reduction was found, on average, to be 2.16 log/CFU mL after treatment and 3.06 log after 24 h. The ANOVA test showed that this treatment was significantly (*p*-value < 0.05) distinguishable from the control group. The Tukey test supported the assumption that the applied ultrasound power and treatment duration have a significant effect (*p*-value: 0.001) on the decrease in the *Salmonella* count, compared to the control group.

**Figure 2:**
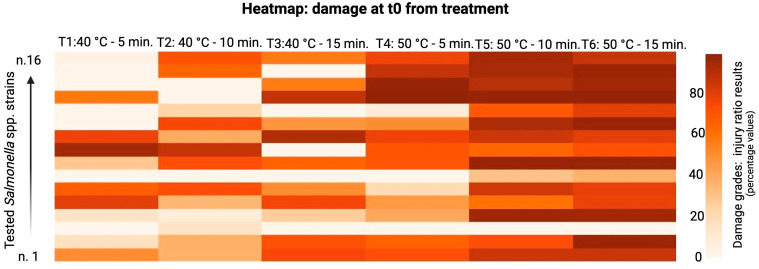
Damage after treatment. T1: 40 °C 5’ treatment; T2: 40 °C 10’ treatment; T3: 40 °C 15’ treatment; T4: 50 °C 5’ treatment; T5: 50 °C 10’ treatment; T6: 50 °C 15’ treatment.

**Figure 3:**
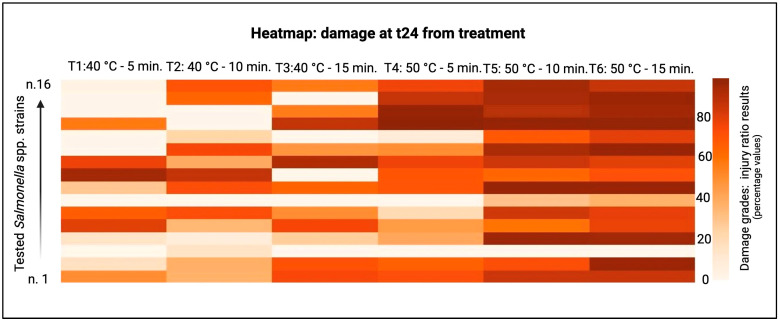
Damage 24 h after treatment. T1: 40 °C 5’ treatment; T2: 40 °C 10’ treatment; T3: 40 °C 15’ treatment; T4: 50 °C 5’ treatment; T5: 50 °C 10’ treatment; T6: 50 °C 15’ treatment.

The sublethal injured cells, and their relative injury ratios, were estimated by the difference in the number of CFUs obtained after plating treated cells in the non-selective medium and the selective medium, as shown in the following heatmaps (see Figure 2 and Figure 3). The major damage was observed at T2 coupled with 10 min of treatment and T2 at 15 min. There was no damage variability during the storage time.

The range of response values at t24 was calculated and interpreted, as previously described in the Section 2. A detailed illustration of the obtained results is schematically reported in Table 2.

The calculated injury ratios, as described before, permitted us to obtain average values of 53.66% at t0 and 53.12% at t24 as general expressions, considering all time–temperature exposures. A detailed representation is provided in Table 3.

It is also important to mention that the highest obtained injury ratios (values more than 90.0%) were observed in the prolonged exposures for 15 min at both T1 and T2. On the contrary, 2/16 (12.5%) *Salmonella* strains (with special regard to the ID: 117 and 685, as reported in Table 3) were not lesioned by the treatments of T1 and T2 at stage t0. It is also mandatory to report that these mentioned strains showed injuries at step t24.

## 4. Discussion

Among innovative food technologies, focused ultrasounds have acquired consistent attention due to their sanitation ability on food matrices determining shelf-life prolongation and also guaranteeing safe products to the final consumers [3]. Among the strict bacterial pathogens, *Escherichia coli* and *Staphylococcus aureus* (also included in the EU Reg. No. 2073/2005 about the so-called *food safety criteria* and *food hygiene process*) have been tested (performing in vitro investigations) using the focused ultrasound exposure, as described by Li et al. [16].

The used settings of 800 W/40 kHz/100% amplitude [with similar ratio time–temperature, as described by Li et al. [16] were applied in the present study based on the discovered efficacy observed in the scientific literature. Following this rationale, *Salmonella* spp. were marginally studied to investigate the bacterial sublethal or lethal effects of the thermosonication method, and for this reason, the obtained original data describe promising applicability on the selected genus. Indeed, all treated strains presented high susceptibility at stage t24, where the cell counts reduced to 1.5–2.0 log CFU/mL from the initial concentration (5.0 log CFU/mL), as graphically represented in Figure 1 (in the Section 3) at both selected temperatures T1 and T2.

The prolonged exposures for 15 min (both T1 and T2) showed the highest reduction of bacterial loads, in agreement with the results described by Sienkiewicz et al. [21] from *Salmonella enterica* subs. *Typhimurium* strains. It is also important to highlight that none of the research studies have discovered a possible significant statistical correlation between inactivation efficacy and specific bacterial loads. This last affirmation finds a fundamental scientific finding described by any author [16,22] who supposed that focused ultrasounds’ bactericidal effect was independent from the initial load. On the other hand, another proposed scientific opinion was that the mechanical and chemical energy, generated by ultrasonic waves, results in high bacterial concentrations [22]. Conversely, Li et al. [16] suggested that high concentrated bacterial solutions determine aggregation, providing resistant physical forms to the ultrasound exposures; indeed, these forms (composed by high concentrated bacteria amounts) permit the creation of a sort of wall which reduces the efficacy of the cavitation phenomenon, and with special regard to the bursting of bubbles on bacteria, if singularly exposed [22].

The performed statistical analysis, the ANOVA test (as described in the Section 3), identified T2 for 15 min as the main efficacious treatment determining a reduction of 2.16 log CFU/mL at t0, gaining a further reduction of 3.06 log CFU/mL at t24. Comparing these results to those in the literature, it is possible to affirm that few scientific investigations have evaluated the focused ultrasound effects and stored samples at refrigerated temperature. In more detail, Bi et al. [23] and Liu et al. [24] confirmed the obtained findings (belonging to the present study), describing a *Salmonella* spp. reduction of 3.31 and 2.0 log CFU/mL, respectively. Conversely, this last-mentioned effect was observed by Valero et al. [25], who reported a bacterial decrement between 1.08 and 1.70 log CFU/mL after 14 days from focused ultrasound exposure. Concerning the bacterial loads’ reductions at t24 of refrigerated storage, it finds the respective scientific explanation to the development of the apoptosis phenomenon that starts, in the treated strains, at t0 and leads to the *exitus* at t24 [24].

It is also important to remark that, in the present scientific investigation, PBS solution was used, as previously performed by Luo et al. [26], which is different from food matrices and may have attenuated the ultrasound effect [26]. As previously described by Lauteri et al. [27], *Salmonella typhimurium* could show a markable variability after treatment and storage. It could be explained by bacterial injury, which is defined as the impact of one or more sublethal treatments on a microorganism [11,28]. In more detail, it is a reversible state in which cells enter in a growth stagnation condition due to structural membrane damage and the expression of specific genes, which incurs functional disorders [28]. However, all samples showed a bacterial count decrement.

The sublethal injured cells were estimated by the difference in the number of CFUs obtained after plating treated cells in the non-selective medium and the selective medium, as shown in the heatmap below (Figure 2 and Figure 3). The major damage was observed in the 50 °C 10 min and 50 °C 15 min treatments. On the contrary, consistent damages were not observed basing on the storage time variable.

At t24 h, there was a remarkable efficacy in all analyzed treatments (i.e., after 24 h, 40 °C 5 min treatment decreased until four times than the evaluation after treatment). Indeed, the focused thermosonication technology causes bacterial wall damage, and after 24 h, there was an increased susceptibility than after treatments, in agreement with the scientific findings proposed by Baumann et al. [29] and Pennisi et al. [30]. The damage of the bacterial cell wall is considered the main mechanism underpinning the ultrasound antimicrobial effect; however, any bacterial strains present resistance to this food technology, and the relative survival mechanisms, defined as the *resistance of ultrasounds* [31], are not well scientifically cleared, as also suggested by Luo et al. [26].

Based on the obtained evidence, *Salmonella* strains were more lethally injured at 50 °C for 15 min (with special regard to t24 more than t0), and from a comparative perspective, a similar pattern was observed and described by Baumann et al. [29] from focused ultrasound-treated *Listeria monocytogenes*.

Concerning *Salmonella* spp., their proper survival mechanisms to ultrasounds have been biochemically explained by Luo et al. [26], who noticed that key enzymes of the tricarboxylic acid cycle were significantly downregulated, which led to a reduced ATP content, although ATPase activity was augmented. *Salmonella* tolerated focused ultrasound stress by upregulating their environmental sensing, chemiotaxis, substance uptake, and ATP production.

## 5. Conclusions

In addition to the microbial stabilization of the thermosonication process, it is also considered a green technology which can provide safe foodstuffs with limited pollution impacts on the environment. Sonication has already been used in food technology processes (extraction, pasteurization, cutting, foaming, drying), and the combined application of the acoustic effect and sublethal temperature has also shown effectiveness in microbial control of food.

Although there is a need to generate more systematic data for a better understanding of different concepts (such as microbial resistance mechanisms, microbial enzyme reactions, and kinetics), the present study contributes to filling gaps of knowledge by highlighting efficacy at specific exposure settings involving *Salmonella typhimurium* strains. Indeed, this in vitro study aims to cover the role of preliminary analyses, which will be applied in food matrices in different physical forms, such as liquid and solid ones. It seeks also to emphasize the impact of focused thermosonication treatments on food quality. Conversely, it is also mandatory to affirm that a careful development and usage in food industries should be considered with special regard to the wide differences of food productions due to their extreme variabilities of ingredients. Therefore, the in vitro studies represent starting points for further studies to provide referring models to gain standardized parameters to be used and successively applied in food industries.

## 6. Patents

Waveco^®^ system (Next Cooking Generation, Milan, Italy)—International Application No.: Patent EP17733039.6.

## Figures and Tables

**Table 1:** *Salmonella typhimurium* strains involved in the present scientific investigation.

Serovar	ID Strain	Origin
*S. typhimurium*	114	Meat product (sausage)
	115	Meat product (sausage)
	117	Meat product (sausage)
	118	Meat product (sausage)
	669	Cecal sample
	670	Pig carcass
	685	Pig carcass
	686	Pig carcass
	687	Cecal sample
	689	Slaughtering environments
	690	Slaughtering environments
	691	Slaughtering environments
	693	Pig carcass
	694	Pig carcass
	695	Pig carcass
	785	Slaughtering environments

**Table 2:** Overview of *Salmonella typhimurium*: a range of responses after 24 h from treatments.

ID	C-T1	r	C-T2	r	C-T3	r	C-T4	r	C-T5	r	C-T6	r
**114**	0.81	R	1.93	I	1.20	I	1.45	I	2.05	S	2.20	S
**115**	0.56	R	1.39	I	1.25	I	1.34	I	1.92	I	4.59	S
**117**	1.71	I	1.90	I	2.16	S	1.98	I	2.06	S	3.96	S
**118**	1.01	I	1.81	I	2.16	S	2.60	S	4.61	S	3.81	S
**669**	0.95	R	1.62	I	0.88	R	1.62	I	1.92	I	2.40	S
**670**	1.44	I	1.15	I	0.89	R	2.96	S	1.45	I	3.39	S
**685**	1.89	I	1.65	I	1.50	I	2.22	S	1.89	I	2.70	S
**686**	1.08	I	1.69	I	1.54	I	2.11	S	2.23	S	2.59	S
**687**	1.41	I	1.07	I	0.57	R	2.92	S	1.30	I	3.22	S
**689**	1.62	I	2.07	S	1.49	I	1.50	I	2.15	S	2.87	S
**690**	0.61	R	0.99	R	1.14	I	2.50	S	3.64	S	2.13	S
**691**	−0.20	R	0.63	R	0.75	R	1.54	I	1.96	I	3.12	S
**693**	0.58	R	1.52	I	1.67	I	1.58	I	2.14	S	2.62	S
**694**	0.49	R	0.56	R	2.24	S	1.01	I	2.54	S	2.74	S
**695**	0.56	R	1.23	I	1.55	I	1.55	I	1.66	I	3.16	S
**785**	2.28	S	2.89	S	1.56	I	3.09	S	2.21	S	3.39	S

C: control; T1: 40 °C 5’ treatment; T2: 40 °C 10’ treatment; T3: 40 °C 15’ treatment; T4: 50 °C 5’ treatment; T5: 50 °C 10’ treatment; T6: 50 °C 15’ treatment; r: gup response; R: resistance; I: intermedium; S: sensible.

**Table 3:** Injury ratio results obtained from the studied *Salmonella typhimurium* strains.

ID Strains	Treatments	SI t0 (%)	SI t24 (%)	ID Strains	Treatments	SI t0 (%)	SI t24 (%)	ID Strains	Treatments	SI t0 (%)	SI t24 (%)
**114**	40 °C–5 min	50.57	71.51	**685**	40 °C–5 min	0.00	30.91	**693**	40 °C–5 min	57.23	72.96
	40 °C–10 min	37.16	87.05		40 °C–10 min	0.00	24.68		40 °C–10 min	0.00	50.52
	40 °C–15 min	74.37	99.42		40 °C–15 min	0.00	2.36		40 °C–15 min	86.29	42.59
	50 °C–5 min	72.06	0.00		50 °C–5 min	0.00	33.33		50 °C–5 min	99.49	88.94
	50 °C–10 min	84.36	0.00		50 °C–10 min	30.85	34.33		50 °C–10 min	98.28	95.04
	50 °C–15 min	84.85	90.78		50 °C–15 min	0.00	96.15		50 °C–15 min	98.86	98.67
**115**	40 °C–5 min	16.33	66.67	**686**	40 °C–5 min	28.95	0.00	**694**	40 °C–5 min	0.00	0.00
	40 °C–10 min	37.50	75.00		40 °C–10 min	72.68	0.00		40 °C–10 min	0.00	71.43
	40 °C–15 min	71.05	48.78		40 °C–15 min	65.38	50.00		40 °C–15 min	56.14	0.00
	50 °C–5 min	65.71	60.42		50 °C–5 min	69.47	93.44		50 °C–5 min	98.31	77.97
	50 °C–10 min	72.73	0.00		50 °C–10 min	97.62	85.19		50 °C–10 min	89.47	90.00
	50 °C–15 min	97.22	75.00		50 °C–15 min	98.11	94.12		50 °C–15 min	95.24	83.33
**117**	40 °C–5 min	0.00	81.01	**687**	40 °C–5 min	94.68	87.00	**695**	40 °C–5 min	0.00	58.18
	40 °C–10 min	14.95	98.81		40 °C–10 min	86.11	58.14		40 °C–10 min	64.35	0.00
	40 °C–15 min	0.00	6.45		40 °C–15 min	0.00	77.22		40 °C–15 min	0.00	44.74
	50 °C–5 min	0.00	57.58		50 °C–5 min	68.83	95.00		50 °C–5 min	86.11	90.00
	50 °C–10 min	0.00	50.00		50 °C–10 min	63.64	95.35		50 °C–10 min	93.75	94.12
	50 °C–15 min	0.00	0.00		50 °C–15 min	71.43	92.86		50 °C–15 min	97.56	0.00
**118**	40 °C–5 min	14.04	26.32	**689**	40 °C–5 min	77.17	91.67	**785**	40 °C–5 min	2.44	48.15
	40 °C–10 min	8.33	84.00		40 °C–10 min	39.47	0.00		40 °C–10 min	70.97	0.00
	40 °C–15 min	26.47	95.45		40 °C–15 min	90.83	0.00		40 °C–15 min	57.14	0.00
	50 °C–5 min	40.74	0.00		50 °C–5 min	76.97	96.00		50 °C–5 min	76.27	94.68
	50 °C–10 min	96.43	0.00		50 °C–10 min	84.17	88.00		50 °C–10 min	94.29	31.58
	50 °C–15 min	95.24	90.91		50 °C–15 min	79.41	91.67		50 °C–15 min	85.71	66.67
**669**	40 °C–5 min	79.40	98.25	**690**	40 °C–5 min	0.00	0.00				
	40 °C–10 min	34.84	60.87		40 °C–10 min	74.92	0.00				
	40 °C–15 min	75.44	70.83		40 °C–15 min	47.25	88.24				
	50 °C–5 min	45.77	0.00		50 °C–5 min	50.24	64.09				
	50 °C–10 min	59.93	99.04		50 °C–10 min	92.37	97.58				
	50 °C–15 min	77.50	0.00		50 °C–15 min	97.87	50.00				
**670**	40 °C–5 min	67.09	0.00	**691**	40 °C–5 min	0.00	0.00				
	40 °C–10 min	72.58	71.74		40 °C–10 min	23.08	0.00				
	40 °C–15 min	50.56	0.00		40 °C–15 min	0.00	82.08				
	50 °C–5 min	20.00	0.00		50 °C–5 min	9.09	0.00				
	50 °C–10 min	83.72	94.12		50 °C–10 min	68.66	56.72				
	50 °C–15 min	78.57	85.29		50 °C–15 min	79.17	98.89				

SI: sublethal injury; t0: plating immediately after treatment; t24: plating after 24 h.

## Data Availability

The original contributions presented in the study are included in the article, further inquiries can be directed to the corresponding author.
